# Ion-Induced Lateral Damage in the Focused Ion Beam Patterning of Topological Insulator Bi_2_Se_3_ Thin Films

**DOI:** 10.3390/ma16062244

**Published:** 2023-03-10

**Authors:** Rubén Gracia-Abad, Soraya Sangiao, Sandeep Kumar Chaluvadi, Pasquale Orgiani, José María De Teresa

**Affiliations:** 1 Instituto de Nanociencia y Materiales de Aragón (INMA), CSIC-Universidad de Zaragoza, 50009 Zaragoza, Spain; 2 Laboratorio de Microscopías Avanzadas (LMA), Universidad de Zaragoza, 50018 Zaragoza, Spain; 3 Departamento de Física de la Materia Condensada, Universidad de Zaragoza, 50009 Zaragoza, Spain; 4 CNR-IOM, TASC Laboratory in Area Science Park, 34149 Trieste, Italy

**Keywords:** topological insulator, Bi_2_Se_3_, focused ion beam, patterning, irradiation damage

## Abstract

Focused Ion Beam patterning has become a widely applied technique in the last few decades in the micro- and nanofabrication of quantum materials, representing an important advantage in terms of resolution and versatility. However, ion irradiation can trigger undesired effects on the target material, most of them related to the damage created by the impinging ions that can severely affect the crystallinity of the sample, compromising the application of Focused Ion Beam to the fabrication of micro- and nanosized systems. We focus here on the case of Bi_2_Se_3_, a topological material whose unique properties rely on its crystallinity. In order to study the effects of ion irradiation on the structure of Bi_2_Se_3_, we irradiated with Ga^+^ ions the full width of Hall-bar devices made from thin films of this material, with the purpose of inducing changes in the electrical resistance and characterizing the damage created during the process. The results indicate that a relatively high ion dose is necessary to introduce significant changes in the conduction. This ion dose creates medium-range lateral damage in the structure, manifested through the formation of an amorphous region that can extend laterally up to few hundreds of nanometers beyond the irradiated area. This amorphous material is no longer expected to behave as intrinsic Bi_2_Se_3_, indicating a spatial limitation for the devices fabricated through this technique.

## 1. Introduction

During the last few decades, advances in miniaturization techniques, along with the requirements of size and shape of new devices, have allowed for probing the limits of the available lithography techniques. In particular, the use of Focused Ion Beam (FIB) has gained importance as a versatile technique that allows one to structure matter at the submicrometer scale, providing high control over the final shape and geometry of the devices [1]. This makes it ideal for the investigation of emerging materials in condensed matter, offering new possibilities for their manipulation and the investigation of unconventional behaviors [2].

The most straightforward application of FIB is direct milling [3,4]. In this process, the ions are accelerated and focused to a nanometric spot on a target sample, impinging on its surface. If the collisions are energetic enough, this enables material removal and patterning of extremely fine structures. However, FIB milling may induce uncontrollable damage coming from colliding ions, secondary processes, or heating effects. The consequences of these events depend on different factors, such as the target material or the working parameters, and the range of damage can go from a few tens to several hundred nanometers, considerably reducing the amount of functional material and significantly affecting the lateral resolution of the patterning. This can represent a serious limitation in certain cases [5]. In addition, in nanosystems, the range of damage can be comparable to the size of the system, significantly altering their properties [6]. On the other hand, in many materials, preserving the properties of their surfaces is crucial so that they show their most intriguing characteristics.

Nowadays, topological materials are a hot topic in the field of condensed matter physics. Specifically, topological insulators (TI) have attracted much attention due to their potential application in quantum computing and spintronics [7]. These materials behave as insulators in their interior, whereas they support topologically protected metallic surface states. Notably, Bi_2_Se_3_ is one of the most studied TIs due to its simple surface band structure, containing a single Dirac cone, and its relatively large bandgap that should make the topological regime in which the bulk states are suppressed more accessible [8].

Several techniques have been applied for the patterning of Bi_2_Se_3_ thin films so far, UV lithography being the most widely used [9,10,11]. Regarding the fabrication of contacts, Electron Beam Lithography (EBL) has also been employed [12,13], avoiding the use of masks present in UV lithography, and simplifying the process. However, both require the use of chemical resists and multiple steps [14]. In this sense, FIB patterning represents an advantage since it is resist-free, which avoids possible chemical contamination or degradation of the surface. Besides, it can be implemented in a single quick step, and it offers the ability to customize the final design [2]. Nevertheless, the unique properties of this material are highly dependent on its crystallinity as well as on the quality of the interfaces, and these factors can be altered by ion irradiation. This is the reason why a deep understanding of such effects is necessary. FIB patterning has already been applied on Bi_2_Se_3_ for the fabrication of nanowires [15,16] and even damage has been considered in some studies [17]. Here, the authors demonstrated the good capabilities of FIB for shaping and thinning Bi_2_Se_3_ flakes, and they also reported the creation of amorphous regions in the material, as well as the formation of Se-deficient areas, that can also affect transport. All these effects established a limitation for the fabrication of high-quality nanowires down to a width of 150 nm. However, a systematic analysis of FIB-induced damage in Bi_2_Se_3_ thin films with different thicknesses showing a detailed structure characterization of that damage is lacking.

Herein, we investigate the effects of using FIB on Bi_2_Se_3_ thin films in order to establish the possible limitations of this technique. We first irradiate Hall-bar systems made out of Bi_2_Se_3_ films and check how their electrical resistance changes. Subsequently, the effects produced on the crystal structure are characterized. We will show that the damage presents a medium-range character, extending laterally up to several hundred nanometers beyond the point of impact. Such an undesired effect should be taken into account when fabricating Bi_2_Se_3_-based devices at the submicrometer scale by FIB.

## 2. Materials and Methods

For the purpose of this work, several 4 μm-wide Hall-bar devices were prepared by optical lithography from Bi_2_Se_3_ thin films of 12.5, 40, 45, 52, and 55 nm thickness (information for the 45 nm sample is used in the Supplementary File) [18,19,20]. The films were grown by Pulsed Laser Deposition (PLD) on sapphire (001) substrates. Their crystallinity was characterized by X-ray Diffraction (XRD), whereas the presence of topological surface states was validated by Angle-Resolved Photoemission Spectroscopy (ARPES). The lithography process was carried out in an MA6 mask-aligner from SUSS MICROTEC (Garching bei München, Germany) following a two-step process, with a first standard etching process to define the Hall bar, and a second lift-off process in order to define the electrical contacts. For the etching, an ion milling machine model 600 from SISTEC (Chemnitz, Germany) was used, whereas an Auto500 e-beam evaporator from BOC Edwards (Burgess Hill, UK) was employed for the growth of the gold contacts.

**Figure 1:**
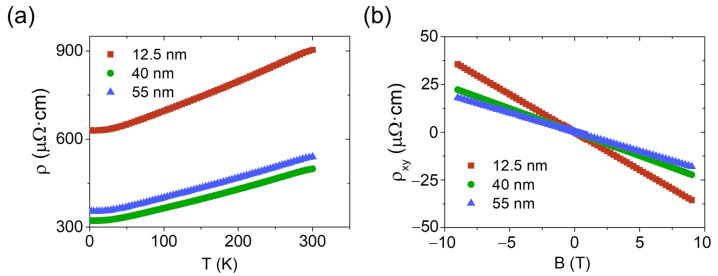
Electrical measurements of Bi_2_Se_3_ samples with thicknesses of 12.5, 40, and 55 nm: (**a**) Thermal dependence of the longitudinal resistivity. (**b**) Magnetic field dependence of the Hall resistivity measured at 2 K.

The electrical properties of the films were characterized by magnetotransport measurements in a Physical Properties Measurement System (PPMS) from Quantum Design (San Diego, CA USA), covering temperatures down to 2 K and perpendicular magnetic fields up to 9 T. The longitudinal resistivity versus temperature curve shows a metallic behavior (Figure 1a), whereas the magnetic dependence of the Hall resistivity determines that the transport is n-type and is attributed to a single type of carrier (Figure 1b). This has been previously reported in pure Bi_2_Se_3_ and is ascribed to the natural formation of Se vacancies in the material that act as donor impurities, populating the bulk conduction band, and hindering the isolation of the topological surface states. Hall resistivity measurements allow us to determine a sheet carrier density and a mobility in the range of (1.8–31.5) × 10^13^ cm^−2^ and 50.3–77.5 cm^2^/(*V* · *s*), respectively, indicating a strong contribution to transport from the bulk states in these samples [21,22,23].

**Figure 2:**
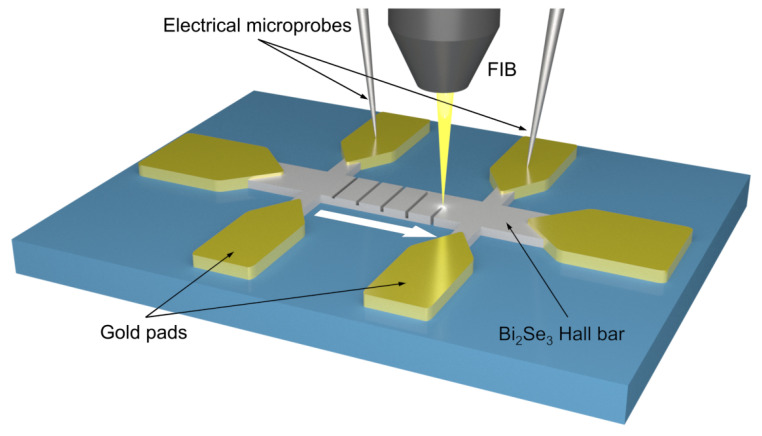
Sketch of the irradiation process of a working region. The white arrow indicates the sense of increasing irradiation dose, as indicated below in Figure 3b.

**Figure 3:**
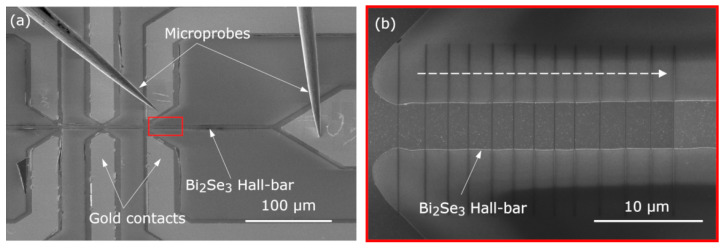
Scanning Electron Microscopy (SEM) images showing: (**a**) microprobes configuration for the in situ electrical measurements. (**b**) Magnified view of the region marked with a red rectangle in (**a**). FIB-irradiated line patterns through the entire width in a working region of a Hall-bar device. The dashed white arrow indicates the sense of increasing ion dose.

The Hall bars were irradiated across their full width using line patterns. Each irradiation process was carried out in a working region, which is defined as the region in the Hall bar between two consecutive contacts. Inside the region, a series of lines made with an increasing ion dose are performed in order to induce changes in its electrical resistance (see Figure 2 and Figure 3), looking for the minimum dose necessary to produce significant changes in the electrical response of the system. For the irradiation process, a Dual Beam Helios Nanolab 650 FIB-SEM equipment from Thermofisher (Hillsboro, OR, USA) was used. This equipment combines a vertical field-emission electron column with a 52^∘^ tilted Ga-based ion column, both with a maximum acceleration voltage of 30 kV, which allows one to simultaneously fabricate and image the process. Both beams intersect at the eucentric point, which is placed 4.15 mm below the pole of the electron column. The accelerating voltage and current of the ion column were 30 kV and 1.1 pA, respectively. A dwell time of 1 μs and a beam spot overlap of 50% were chosen. The ion dose was progressively increased by simply increasing the irradiation time.

Electrical resistance was extracted from I-V curves taken in situ after each irradiation line pattern, using electrical microprobes from Kleindiek Nanotechnik GmbH (Reutlingen, Germany) and a 6221 DC current source/2182A nanovoltmeter from Keithley Instruments (Cleveland, OH, USA), with a two-probe measurement configuration by injecting a DC current and measuring the voltage drop simultaneously (Figure 3a). Contact resistance was negligible in this case (order of magnitude of few Ω) compared to device resistance (order of magnitude of few kΩ), and since we are interested in resistance changes and not in absolute values of the resistance, four-probe measurements were not required.

For structural characterization, some lamellas of the irradiated regions were prepared and observed in a Titan Cube Transmission Electron Microscope from Thermofisher (Hillsboro, OR, USA) with High-Resolution Transmission Electron Microscopy (HRTEM) capabilities. This equipment provides atomic resolution of the lattice structure, enabling examination of the structural effects caused by ion irradiation in the sample and the determination of the extent of damage.

## 3. Results

**Figure 4:**
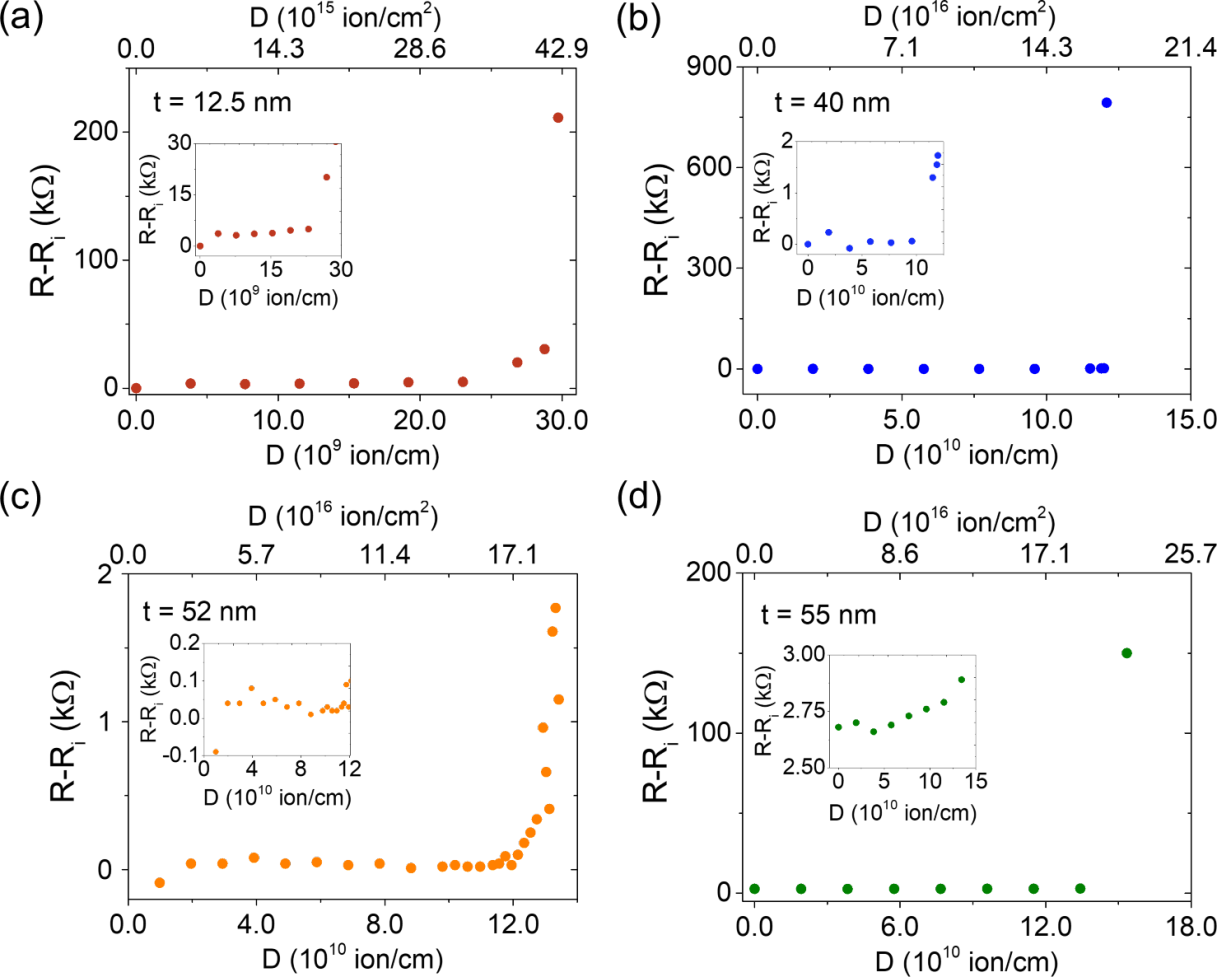
Change in electrical resistance $R-R_{i}$, calculated as the difference between the resistance after (*R*) and before ($R_{i}$) irradiating a line with a given dose value (*D*), as a function of the ion dose for samples with four different thicknesses: 12.5 (**a**), 40 (**b**), 52 (**c**), and 55 nm (**d**). In the lower x-axis, the dose is expressed in ion/cm, whereas in the upper one, it is expressed in ion/cm^2^, considering the beam spot diameter. The insets show the low ion dose region in more detail.

Figure 4 shows the evolution of the change in electrical resistance against the gallium ion dose for the four different film thicknesses. The change in resistance $R-R_{i}$ for a given dose is calculated as the difference between the resistance after (R) and before ($R_{i}$) irradiating a line with that dose. In this way, the increment due only to the last irradiation is obtained. Ion dose is expressed in both ion/cm and in ion/cm^2^. For the latter, a beam spot size of 7 nm provided by the chosen working parameters was considered. For all samples the same behavior was observed: first, a nearly flat region in which the resistance increased slightly. For this range of relatively low doses, only localized amorphization of the structural lattice took place (see the Supplementary File for more details on the evolution of the damage with increasing ion dose). After a certain value of the ion dose, milling started and the amorphization spread beyond the irradiated area. However, no significant changes in the resistance were observed until a higher dose was applied, at which it increased sharply. After this abrupt increase, the electrical resistance increased several orders of magnitude, and the sample could be considered electrically insulating. Thus, the irradiation produces an insulating region in the bar.

In order to compare this feature among the different samples, we can establish a criterion according to which the sample can be considered electrically cut off. This is made to correspond to a value of change of 20 times the initial electrical resistance *R*_0_. The dose corresponding to the irradiation producing such a change is called the critical dose $D_{c}$. The values of $D_{c}$ extracted through this criterion are shown in Table 1. As expected, $D_{c}$ increases with increasing thickness. The value of the present doses is in good agreement with the one found in previous work [16], where an ∼8 nm Bi_2_Se_3_ film with a 20 nm to 30 nm capping layer of Se was irradiated, showing an abrupt increase of the electrical resistance at 3.6 · 10^16^ ion/cm^2^.

**Figure 5:**
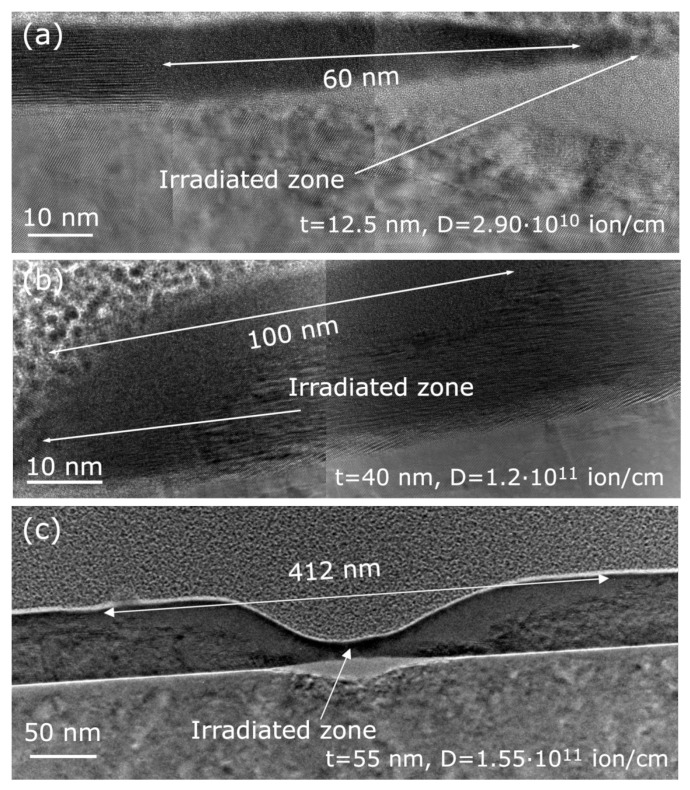
Transmission Electron Microscopy (TEM) images, with white double-headed arrows showing the damage range after high dose irradiation in samples with thicknesses: (**a**) 12.5 nm, (**b**) 40 nm, and (**c**) 55 nm. In (**a**,**b**), just one side of the irradiated zone is shown, whereas in (**c**), both are presented.

To closely examine the effects of the FIB-irradiation process on the samples and to correlate the behavior of the electrical resistance against ion dose with the formation of damage in the crystalline structure, several lamellas corresponding to different doses and thicknesses were prepared and studied by HRTEM (see the Supplementary File for additional images on structural characterization). First, Figure 5 shows the effects of irradiation on three of the four thicknesses for a dose value around $D_{c}$, demonstrating severe lateral damage, reaching from tens of nanometers in the 12.5 nm-thick sample (Figure 5a) up to several hundred nanometers in the 55 nm-thick sample (Figure 5c). In all cases, medium-range damage (>*t*) was observed. This could represent a limitation when applying FIB fabrication to this material, significantly reducing the amount of functional material and representing a constraint for the lateral size of the device. It can be observed that whereas in the thinnest film the damage covers the entire thickness of the film, in the thicker ones, the damage takes place just at the region closest to the film surface. In general, an increase in the range of damage was found with increasing thickness, as expected from the higher dose required for the corresponding irradiation processes.

Regarding the nature of the damage, amorphization took place in the majority of the region. However, this was not happening throughout the affected structure. It was observed in different irradiated areas that right next to the irradiated region, a small area of polycrystalline material was formed. This can be attributed to recrystallization of the region closest to the irradiated area due to local heating that arises from the energy transfer of the ions to the crystal lattice [24,25]. The damage extended throughout the lateral dimension, but in the region closest to the beam incidence point, the temperature was high enough to produce partial recrystallization. The differences in structure can be further confirmed by taking the Fast Fourier Transforms (FFT) of the structure in the TEM images (See Figure S7 in Supplementary File).

**Figure 6:**
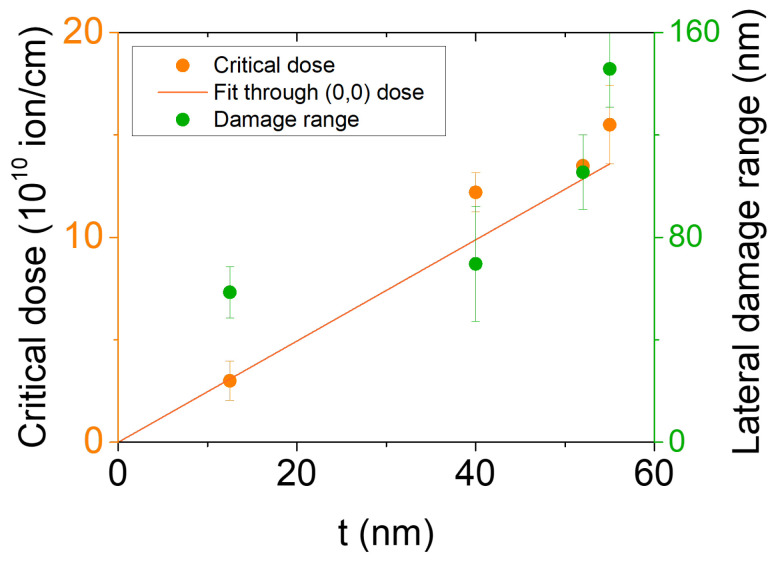
Critical dose $D_{c}$ with linear fitting and lateral damage range plotted against thickness.

As a summary of the observed results, a plot with the values of $D_{c}$ and the lateral damage range against the thickness of the sample is presented in Figure 6. This shows that $D_{c}$ scales almost linearly with thickness. A slight deviation from linearity might be expected due to several factors that participate in the interaction of the ions with the sample, such as local heating or backscattering produced by the substrate, which makes the process more difficult to describe. Indeed, local heating acts as a source of recrystallization. Grain formation after recrystallization might even favor electrical transport through the amorphized areas, delaying the electrical cut. The linear trend was not observed in lateral damage, but a relatively large damage (>*t*) was found in the thinner film of 12.5 nm. In this case, the sapphire substrate can act as a strong source of backscattering that plays an important role in the lateral spreading of the damage. For thicker films, the substrate is not expected to play such a relevant role at the beginning of the irradiation process, given that the ion dose reaching the substrate is much lower. Theoretical or simulation results would be necessary for a more comprehensive understanding of all the complex physical processes involved in the ion irradiation of Bi_2_Se_3_ films.

## 4. Conclusions and Outlook

We have fabricated and ion irradiated Bi_2_Se_3_ patterned films of different thicknesses in order to study the effects of ion irradiation on their electrical resistance and on their crystalline structure. The evolution of the electrical resistance with the increase in ion dose showed initially a weak effect. Only after exceeding a given dose, the electrical resistance changed significantly. This corresponded to the formation of an insulating trench.

In addition, the structural characterization by TEM allowed us to study the range of damage created by the impinging ions. We observed a first stage in which only amorphization took place, remaining localized, followed by a second stage in which milling was triggered, leading to a lateral spreading of the amorphization in the crystalline structure up to several hundred nanometers beyond the irradiated area. This study was carried out under soft working conditions, given that the Ga beam current was set to 1.1 pA, which can be considered a low current corresponding to a short tail in the beam profile.

With these results in mind, we can conclude that there are spatial limitations when directly applying FIB to the fabrication of Bi_2_Se_3_ devices. Specifically, when fabricating devices with dimensions comparable to the extent of the damage, the final performance of the device could be seriously affected. This consideration becomes even more important in thinner samples, where the damage might spread down to the substrate for the entire affected region. Some routes for reducing the damage created by ions have considered the idea of annealing at temperatures around 300 ^∘^C [26]. In other cases, the functional thin films are protected with a sacrificial layer [27]. Other studies have shown that decreasing substrate temperature below room temperature can considerably reduce the amount of damage caused by ion irradiation [5,28,29], which can be an interesting route for further research.

## Figures and Tables

**Table 1:** Values of $D_{c}$ for different thicknesses in ion/cm and ion/cm^2^.

*t* (nm)	$D_{c}$ (10^10^ ion/cm)	$D_{c}$ (10^16^ ion/cm^2^)
12.5	3.0±0.9	4.2±0.8
40	12.2±1.0	17.2±0.9
52	13.5±0.2	19.3±0.2
55	15.5±1.9	21.0±1.6

## Data Availability

The data are available under reasonable request.

## Supplementary Materials

The following supporting information can be downloaded at: https://www.mdpi.com/article/10.3390/ma16062244/s1, Figure S1: Evolution of structural damage with increasing ion dose, Figure S2: Evolution of the I–V characteristics with increasing ion dose, Figure S3: Additional Transmission Electron Microscopy images for the 12.5 nm- thick sample, Figure S4: Additional Transmission Electron Microscopy images for the 40 nm- thick sample, Figure S5: Transmission Electron Microscopy images for the 52 nm- thick sample, Figure S6: Additional Transmission Electron Microscopy images for the 55 nm- thick sample, Figure S7: Transmission Electron Microscopy image with Fast Fourier Transforms of damaged regions. Reference [30] is cited in the Supplementary File.

## References

[1] De Teresa J.M. (2020). Nanofabrication.

[2] Moll P.J. (2018). Focused Ion Beam Microstructuring of Quantum Matter. Annu. Rev. Condens. Matter Phys..

[3] Tseng A.A. (2004). Recent developments in micromilling using focused ion beam technology. J. Micromech. Microeng..

[4] Li P., Chen S., Dai H., Yang Z., Chen Z., Wang Y., Chen Y., Peng W., Shan W., Duan H. (2021). Recent advances in focused ion beam nanofabrication for nanostructures and devices: Fundamentals and applications. Nanoscale.

[5] Schneider M., Gierak J., Marzin J.Y., Gayral B., Gérard J.M. (2000). Focused ion beam patterning of III–V crystals at low temperature: A method for improving the ion-induced defect localization. J. Vac. Sci. Technol. B Microelectron. Nanometer Struct. Process. Meas. Phenom..

[6] Lucot D., Gierak J., Ouerghi A., Bourhis E., Faini G., Mailly D. (2009). Deposition and FIB direct patterning of nanowires and nanorings into suspended sheets of graphene. Microelectron. Eng..

[7] Hasan M.Z., Kane C.L. (2010). Colloquium: Topological insulators. Rev. Mod. Phys..

[8] Xia Y., Qian D., Hsieh D., Wray L., Pal A., Lin H., Bansil A., Grauer D., Hor Y.S., Cava R.J. (2009). Observation of a large-gap topological-insulator class with a single Dirac cone on the surface. Nat. Phys..

[9] Chen J., Qin H.J., Yang F., Liu J., Guan T., Qu F.M., Zhang G.H., Shi J.R., Xie X.C., Yang C.L. (2010). Gate-Voltage Control of Chemical Potential and Weak Antilocalization in *Bi*_2_*Se*_3_. Phys. Rev. Lett..

[10] Zhang G., Qin H., Chen J., He X., Lu L., Li Y., Wu K. (2011). Growth of Topological Insulator *Bi*_2_*Se*_3_ Thin Films on *SrTiO*_3_ with Large Tunability in Chemical Potential. Adv. Funct. Mater..

[11] Wang H., Liu H., Chang C.Z., Zuo H., Zhao Y., Sun Y., Xia Z., He K., Ma X., Xie X.C. (2014). Crossover between Weak Antilocalization and Weak Localization of Bulk States in Ultrathin *Bi*_2_*Se*_3_ Films. Sci. Rep..

[12] Yang F., Qu F., Shen J., Ding Y., Chen J., Ji Z., Liu G., Fan J., Yang C., Fu L. (2012). Proximity-effect-induced superconducting phase in the topological insulator Bi_2_Se_3_. Phys. Rev. B.

[13] Peng H., Lai K., Kong D., Meister S., Chen Y., Qi X.L., Zhang S.C., Shen Z.X., Cui Y. (2010). Aharonov–Bohm interference in topological insulator nanoribbons. Nat. Mater..

[14] Mambakkam S.V., Law S. (2020). Fabrication of topological insulator nanostructures. J. Vac. Sci. Technol. B.

[15] Bhattacharyya B., Sharma A., Awana V.P.S., Srivastava A.K., Senguttuvan T.D., Husale S. (2017). Observation of quantum oscillations in FIB fabricated nanowires of topological insulator (*Bi*_2_*Se*_3_). J. Phys. Condens. Matter.

[16] Fukui N., Hobara R., Hirahara T., Hasegawa S., Miyatake Y., Mizuno H., Sasaki T., Nagamura T. (2014). In situ microfabrication and measurements of *Bi*_2_*Se*_3_ ultrathin films in a multichamber system with a focused ion beam, molecular beam epitaxy, and four-tip scanning tunneling microscope. E-J. Surf. Sci. Nanotechnol..

[17] Friedensen S., Mlack J.T., Drndić M. (2017). Materials analysis and focused ion beam nanofabrication of topological insulator *Bi*_2_*Se*_3_. Sci. Rep..

[18] Orgiani P., Bigi C., Kumar Das P., Fujii J., Ciancio R., Gobaut B., Galdi A., Sacco C., Maritato L., Torelli P. (2017). Structural and electronic properties of *Bi*_2_*Se*_3_ topological insulator thin films grown by pulsed laser deposition. Appl. Phys. Lett..

[19] Bigi C., Orgiani P., Nardi A., Troglia A., Fujii J., Panaccione G., Vobornik I., Rossi G. (2019). Robustness of topological states in *Bi*_2_*Se*_3_ thin film grown by Pulsed Laser Deposition on (001)-oriented *SrTiO*_3_ perovskite. Appl. Surf. Sci..

[20] Gracia-Abad R., Sangiao S., Bigi C., Kumar Chaluvadi S., Orgiani P., De Teresa J.M. (2021). Omnipresence of Weak Antilocalization (WAL) in *Bi*_2_*Se*_3_ Thin Films: A Review on Its Origin. Nanomaterials.

[21] Chen J., He X.Y., Wu K.H., Ji Z.Q., Lu L., Shi J.R., Smet J.H., Li Y.Q. (2011). Tunable surface conductivity in Bi_2_Se_3_ revealed in diffusive electron transport. Phys. Rev. B.

[22] Taskin A.A., Sasaki S., Segawa K., Ando Y. (2012). Manifestation of Topological Protection in Transport Properties of Epitaxial *Bi*_2_*Se*_3_ Thin Films. Phys. Rev. Lett..

[23] Bansal N., Kim Y.S., Brahlek M., Edrey E., Oh S. (2012). Thickness-Independent Transport Channels in Topological Insulator *Bi*_2_*Se*_3_ Thin Films. Phys. Rev. Lett..

[24] Barzola-Quiquia J., Lehmann T., Stiller M., Spemann D., Esquinazi P., Häussler P. (2015). Topological insulator thin films starting from the amorphous phase-*Bi*_2_*Se*_3_ as example. J. Appl. Phys..

[25] Cen X., van Benthem K. (2018). Ion beam heating of kinetically constrained nanomaterials. Ultramicroscopy.

[26] Sharma P.A., Lima Sharma A.L., Hekmaty M., Hattar K., Stavila V., Goeke R., Erickson K., Medlin D.L., Brahlek M., Koirala N. (2014). Ion beam modification of topological insulator bismuth selenide. Appl. Phys. Lett..

[27] Marín L., Morellón L., Algarabel P.A., Rodríguez L.A., Magén C., De Teresa J.M., Ibarra M.R. (2014). Enhanced Magnetotransport in Nanopatterned Manganite Nanowires. Nano Lett..

[28] Gierak J., Ben Assayag G., Schneider M., Vieu C., Marzin J. (1996). 3D defect distribution induced by focused ion beam irradiation at variable temperatures in a GaAsGaAlAs multi quantum well structure. Microelectron. Eng..

[29] Clericò V., Delgado-Notario J.A., Saiz-Bretín M., Malyshev A.V., Meziani Y.M., Hidalgo P., Méndez B., Amado M., Domínguez-Adame F., Diez E. (2019). Quantum nanoconstrictions fabricated by cryo-etching in encapsulated graphene. Sci. Rep..

[30] Corbae P., Ciocys S., Varjas D., Kennedy E., Zeltmann S., Molina-Ruiz M., Griffin S.M., Jozwiak C., Chen Z., Wang L.W. (2023). Observation of spin-momentum locked surface states in amorphous *Bi*_2_*Se*_3_. Nat. Mater..

