# Supplementary material for: Ion-Induced Lateral Damage in the Focused Ion Beam Patterning of Topological Insulator Bi2Se3 Thin Films
Source: Materials (Basel). 2023 Mar 10;16(6):2244. doi: 10.3390/ma16062244 (PMC10051711; doi:10.3390/ma16062244)
Supplement: Supplementary file 1 [file materials-16-02244-s001.zip › materials-2267446-supplementary.pdf]

Supporting Information

for

Ion-induced lateral damage in the Focused Ion Beam patterning of topological insulator Bi_2Se_3 thin films

Rubén Gracia-Abad[1,2,3], Soraya Sangiao[*1,2,3], Sandeep Kumar Chaluvadi[4], Pasquale Orgiani[4] and José María de Teresa[*1,2,3]

Address: [1]Instituto de Nanociencia y Materiales de Aragón (INMA), CSIC-Universidad de Zaragoza, 50009 Zaragoza, Spain; [2]Laboratorio de Microscopías Avanzadas (LMA), Universidad de Zaragoza, 50009 Zaragoza, Spain: Departamento de Física de la Materia Condensada, Universidad de Zaragoza, 50009 Zaragoza, Spain and [4]CNR.IOM, TASC Laboratory in Area Science Park, 34139 Trieste, Italy

Email: Soraya Sangiao – sangiao@unizar.es; José María De Teresa – deteresa@unizar.es

* Corresponding author

- Evolution of damage created by Ga ion irradiation on Bi_2Se_3 thin films

Regarding how the damage is progressively made on the films, we have observed a similar trend in all of our samples. To show this, some data corresponding to a 45 nm-thick sample are exposed in Figure S1. Some lamellas corresponding to doses quite below D_c allow us to understand the first effects of the irradiation. It can be seen that initially the main effects are related just to amorphization of the crystalline structure, with no milling taking place (Figure S1a). The amorphous region is clearly distinguishable from the crystalline planes surrounding it. The colliding ions are energetic enough to produce damage down to the substrate, but the dose is not high enough to sputter the surface atoms out of the film. Even though the resistance is just slightly higher after irradiation, amorphous Bi_2Se_3 is much more resistive than crystalline Bi_2Se_3, as can be verified by considering the resistance increment after irradiation and a length of the amorphous trench in the order of tens of nanometers. With these values, together with the width and the thickness of the Hall bar, a resistivity in the order of $10000\ \mu\Omega \cdot cm$ is obtained, in agreement with the reported values of amorphous Bi_2Se_3 [1], and much higher than that of crystalline Bi_2Se_3, in the order of $100\ \mu\Omega \cdot cm$.

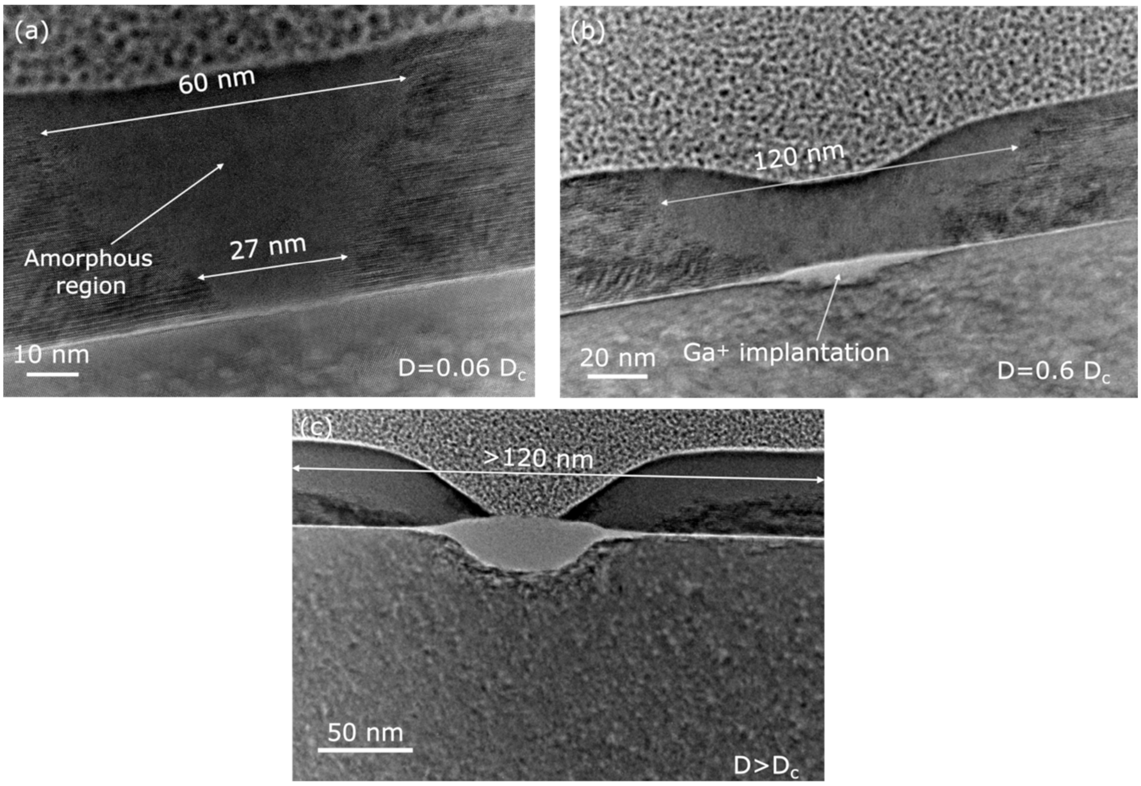

Figure S1: TEM images showing the evolution of damage in a 45-nm thick sample (a) At lower doses ($D \ll D_c$) only amorphization takes place. (b) At higher doses ($D \sim 0.6 D_c$), milling is triggered and Ga implantation into the substrate takes place. (c) Above D_c the milling penetrates down to the substrate giving rise to an insulating trench.

Figure S2 shows the I-V characteristics for different values of the FIB-irradiation dose relative to D_c showing an increase of the slope with increasing dose. Above a certain dose, milling is triggered, and it continues until the substrate is reached (Figure S1b,c). At this point, an insulating trench is obtained, and Rdiverges. It can be observed how the amorphous region spreads over distances far from the irradiated spot, reaching several hundreds of nanometers. The electrical isolation corresponding to D_c takes place slightly below this point. Taking this into account, it looks like a big amount of damage is necessary to make the material insulating. It can also be noticed that before reaching the substrate, Ga ions start implanting into the substrate, giving rise to bumps at its surface. These bumps can be responsible for some deformation in the regions of the film close to the irradiation, even more pronounced in the case of thinner films.

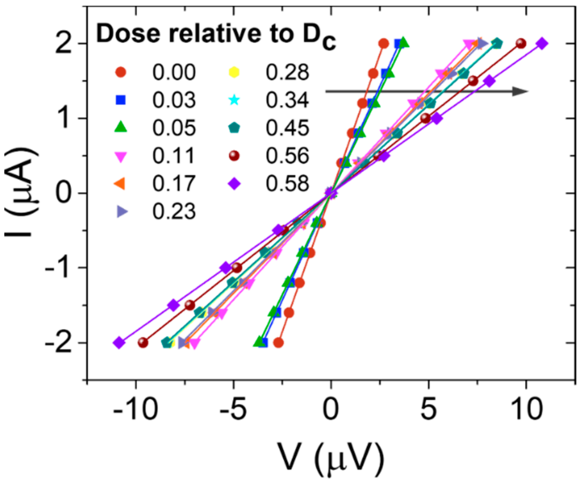

Figure S2: Evolution of the I-V curve of a 45 nm-thick sample with the ionic dose. The arrow indicates the sense of increasing resistance.

- Structural characterization of ion irradiated Bi_2Se_3 thin films:

The following images show structural characterization carried out through Transmission Electron Microscopy (TEM) in the four samples referred to in the main text with thicknesses 12.5, 40, 52 and 55 nm. All of them show regions irradiated with doses in the range of ±5% of the corresponding D_c indicated in the main text. The lateral damage range is indicated in the figures.

(a)

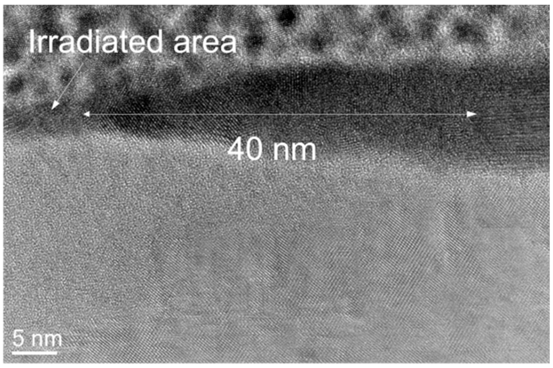

(b)

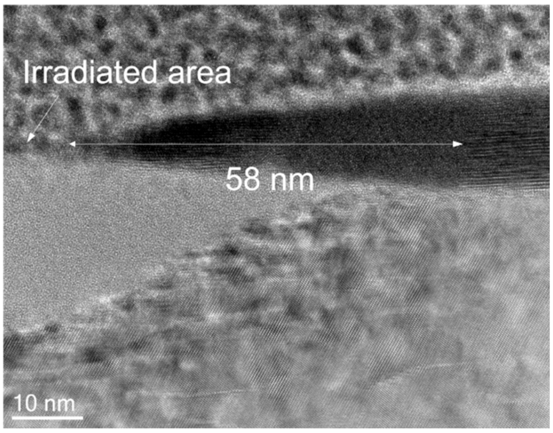

Figure S3: TEM images of two regions irradiated with different doses in the 12.5 nm-thick sample: (a) $D = 2.30 \times 10^{10}\ ion/cm$, (b) $D = 2.68 \times 10^{10}\ ion/cm$.

(a)

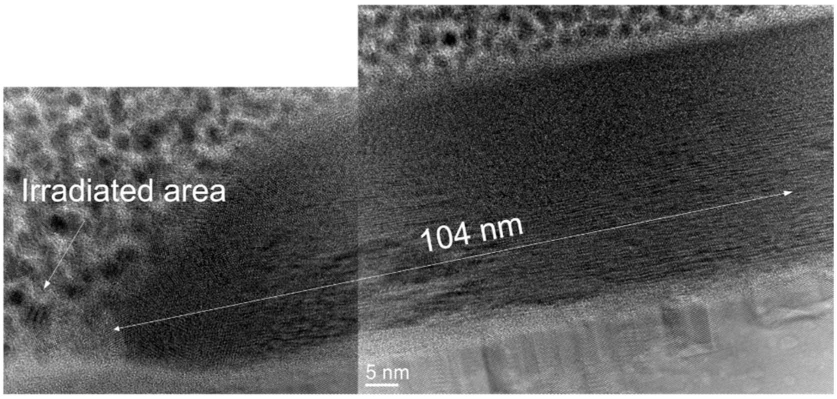

(b)

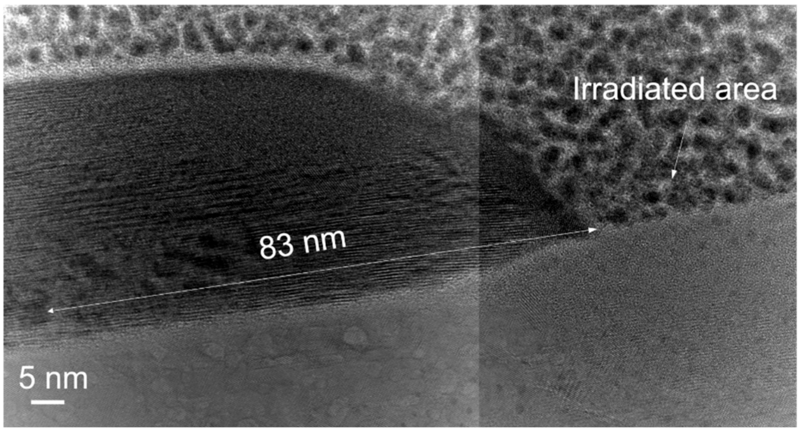

Figure S4: TEM images of two regions irradiated with different doses in the 40 nm-thick sample: (a) $D = 1.15 \times 10^{11}\ ion/cm$, (b) $D = 1.19 \times 10^{11}\ ion/cm$.

(a) (b)

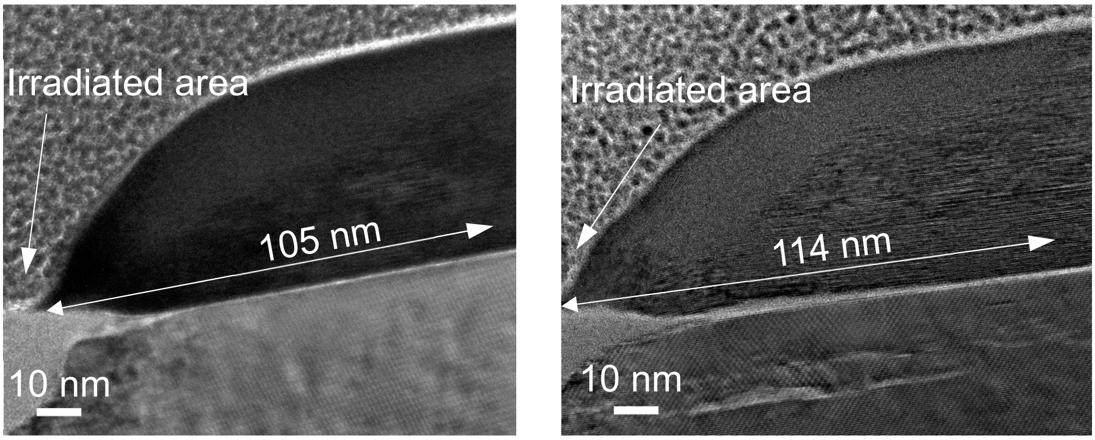

Figure S5: TEM images of two regions irradiated with different doses in the 52 nm-thick sample: (a) $D = 1.21 \times 10^{11}\ ion/cm$, (b) $D = 1.29 \times 10^{11}\ ion/cm$..

(a)

(b)

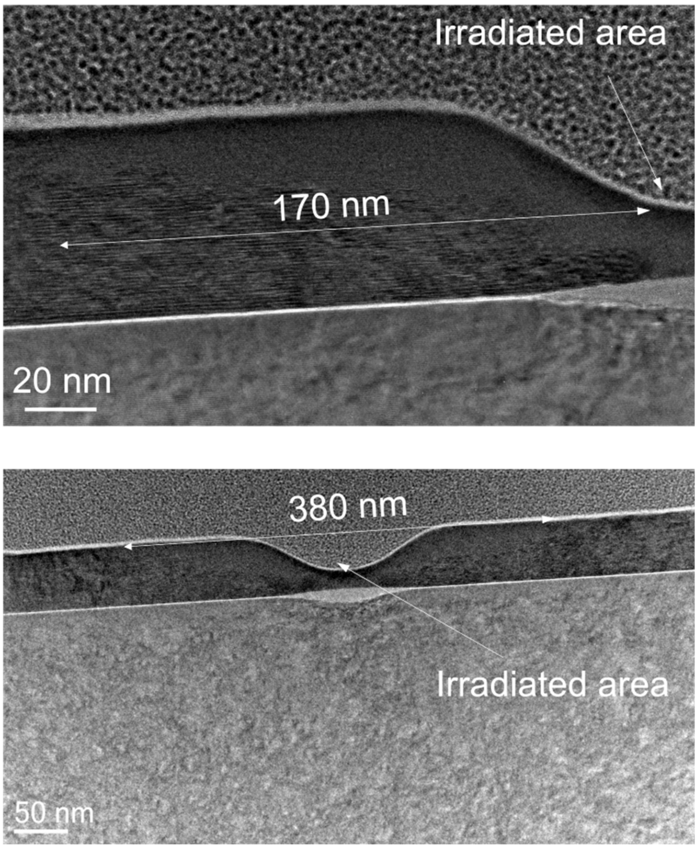

Figure S6: TEM images of two regions irradiated with different doses in the 55 nm-thick sample: (a) $D = 1.15 \times 10^{11}\ ion/cm$, (b) $D = 1.34 \times 10^{11}\ ion/cm$.

Figure S7 shows the Fast Fourier Transform (FFT) of different regions next to an irradiated area of a 52 nm-thick film. These FFTs provide a representation of the crystal

lattice in the reciprocal space, i.e., the spots in the FFT represent a periodicity present in the real space image of the region. Considering this, only region number 1, with a series of spots in the FFT presents a periodicity in the real space image. Specifically, the spots superimposed to a blurred background, indicate a polycrystalline region. In regions number 2 and 3, the presence of blurred circles with no spots indicates complete amorphization.

The presence of a polycrystalline region right next to the irradiated area is likely to be attributed to a reheating of the area caused by the irradiation process.

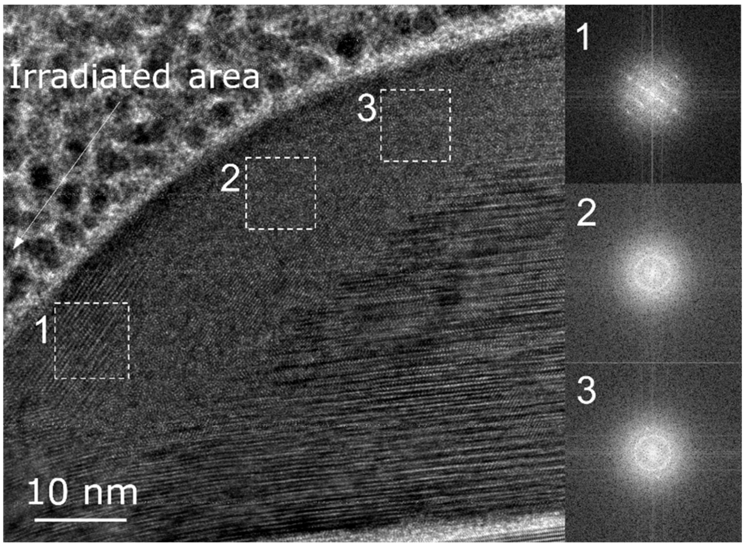

Figure S7: TEM image of an irradiated region with FFT of three different affected regions.

References

Corbae P, Ciocys S, Varjas D, Kennedy E, Zeltmann S, Molina-Ruiz M, Griffin SM, Jozwiak C, Chen Z, Wang LW, Minor AM, Scott M, Grushin AG, Lanzara A, Hellman F. Observation of spin-momentum locked surface states in amorphous Bi_2Se_3. Nat Mater. 2023 Feb;22(2):200-206. doi: 10.1038/s41563-022-01458-0. Epub **2023**. PMID: 36646794.
